# Direct numerical solutions of the SIR and SEIR models via the Dirichlet series approach

**DOI:** 10.1371/journal.pone.0287556

**Published:** 2023-06-30

**Authors:** Kiattisak Prathom, Asama Jampeepan

**Affiliations:** Division of Mathematics and Statistics, Walailak University, Nakhon Si Thammarat, Thailand; University of the Philippines Diliman, PHILIPPINES

## Abstract

Compartment models are implemented to understand the dynamic of a system. To analyze the models, a numerical tool is required. This manuscript presents an alternative numerical tool for the SIR and SEIR models. The same idea could be applied to other compartment models. The result starts with transforming the SIR model to an equivalent differential equation. The Dirichlet series satisfying the differential equation leads to an alternative numerical method to obtain the model’s solutions. The derived Dirichlet solution not only matches the numerical solution obtained by the fourth-order Runge-Kutta method (RK-4), but it also carries the long-run behavior of the system. The SIR solutions obtained by the RK-4 method, an approximated analytical solution, and the Dirichlet series approximants are graphically compared. The Dirichlet series approximants order 15 and the RK-4 method are almost perfectly matched with the mean square error less than 2 × 10^−5^. A specific Dirichlet series is considered in the case of the SEIR model. The process to obtain a numerical solution is done in the similar way. The graphical comparisons of the solutions achieved by the Dirichlet series approximants order 20 and the RK-4 method show that both methods produce almost the same solution. The mean square errors of the Dirichlet series approximants order 20 in this case are less than 1.2 × 10^−4^.

## Introduction

The SIR model is a simple system of nonlinear differential equations that has a rich dynamic. This model is an example of compartment models mainly studied in epidemiology [1–3]. The SIR model and its adaptive versions have been used broadly to investigate the dynamic of a considered system. The models are applicable in public health to intervene and control a disease’s transmission [1, 3–12]. Once a compartment model appears, a tool is required to analyze the model. Understanding the flow of the model would lead the researchers to an assumption to control the disease spreading. There are several tools available to handle the compartment models depending on the situation we are dealing with. Some researchers have sometimes used a computer model to assess certain situations [13, 14]. Others have implemented a numerical method to investigate the models. Since computer models can be utilized only in some circumstances, a numerical method would be an alternative tool. Some recent studies of the compartment models have focused on the fractional-order compartment models by considering the models under the fractional-order *α*, where *α* ∈ (0, 1). As *α* approaches 1, the models become the classical compartment models. The fractional-order SIR model has been demonstrated to have biological significance when *α* ≥ 0.8 [15].

The Dirichlet series is one of the interesting well-known series in the branch of complex analysis since it is a convergent series. The general Dirichlet series is in the form

$$\sum_{i=0}^{\infty }a_{i}e^{-\lambda_{i}t},$$

where 0 = λ_0_ < λ_1_ < … and $a_{i}\in C\backslash \left\{0\right\}$. The started term *a*_0_ is the approached value of the series as *t* goes to infinity. The Dirichlet series solution satisfying some specific differential equations has been studied in [16–18]. Investigating the Dirichlet series satisfying the compartment models would lead us to a new method to obtain the models’ solution which will be mainly discussed in this paper.

This manuscript considers the classical SIR and SEIR models which are equivalent to the fractional-order *α* when *α* approaches one. The existence and uniqueness of the models’ solutions are confirmed by a well-known theorem appearing in many differential equations books. However, their exact non-parametric solutions, or what we can call the closed-form formulas have been unknown. The results in [19] have illustrated the exact analytical solution of the SIR model in the parametric form. Although the parametric analytical solution has been derived, a numerical method is still required to obtain the approximate solutions *S*(*t*), *I*(*t*), *R*(*t*) at any time *t*. A well-known numerical method to obtain an approximate solution of any compartment model is the fourth-order Runge-Kutta method (RK-4) which is an iterative method depending on a given step size and it does not provide the long-run behavior of the solution. An approximate closed-form solution on the other hand may give the long-run behavior with no step size needed. This rises up researchers’ interest to derive an approximate closed-form solution of the SIR and its related models. Some approximate closed-form solutions of the SIR model have been studied in [20–23]. Considering the solution in the form of a power series expression is one simple way to obtain an approximate closed-form solution of a nonlinear system of differential equations. However, the power series solution as a function of time *t* diverges as *t* approaches infinity. The power series solution of the SIR model has been studied by [24]. A multistage technique repeating the same order of power series approximation with updated initial conditions is a method to get a numerical solution of the SIR model [25]. Using power series approximation or repeatedly using a fixed order of power series approximation can not eventually reach the long-run behavior of the system due to the divergence of the power series. A power series approach together with a defined approximant is presented in [20]. Padé approximant are the other two numerical methods that can lead the approximated solution to the long-run behavior [26, 27]. Considering the Dirichlet series solution would be an alternative path to overcome the limitation mentioned above.

## SIR model

In the SIR model, the rates that the proportions of susceptible *S*, infectious *I*, and recovered *R* change over time are described by

$$\begin{array}{cc} \frac{dS}{dt} & =-\beta IS, \end{array}$$ (1)

$$\begin{array}{cc} \frac{dI}{dt} & =\beta IS-\gamma I, \end{array}$$ (2)

$$\begin{array}{cc} \frac{dR}{dt} & =\gamma I, \end{array}$$ (3)

where the initial conditions are assumed to be *S*(0) = *S*_0_ > 0, *I*(0) = *I*_0_ > 0, *R*(0) = 0, and $\beta ,\gamma \in R_{0}^{+}$. Another notation used in this model is

$$\begin{array}{c} S_{\infty }:=\underset{t\to \infty }{\text{lim}}S\left(t\right). \end{array}$$

Note that the solution functions *S*, *I*, *R* are single variable real-valued functions that $S,I,R:R_{0}^{+}\to \left[0,1\right]$. In addition, the total proportion at any time *t* is assumed to satisfy *S*(*t*) + *I*(*t*) + *R*(*t*) = 1.

Eqs (1)-(3) can be combined to be in the form of a Bernoulli differential equation presented in [19, 21]. Solving the Bernoulli differential equation leads to an approximate analytical solution of the model. Here, we present an approach to derive the approximate analytical solution without transforming Eqs (1)-(3) into the Bernoulli differential equation. A result derived from this approach will be used further.

We first rewrite (1) as

$$\begin{array}{c} I=-\frac{1}{\beta }(\frac{S^{\prime }}{S}). \end{array}$$ (4)

When we substitute this equation in (2), we get

$$\begin{array}{c} I^{\prime }=-(\beta S-\gamma )(\frac{1}{\beta })(\frac{S^{\prime }}{S}). \end{array}$$ (5)

Differentiating both sides of (4) with respect to *t* and then equating with (5), we have

$$\begin{array}{c} SS^{\prime \prime }-S^{\prime 2}-(\beta S-\gamma )SS^{\prime }=0. \end{array}$$ (6)

The solution of this differential equation can be found by defining the function *ϕ* as

$$\begin{array}{c} \frac{1}{\phi }:=\frac{1}{S}\frac{dS}{dt}=\frac{d}{dt}\;\text{ln}\;S. \end{array}$$ (7)

Note that our approach of defining *ϕ* above is different from the approach presented in [19]. After using the notation of *ϕ* with (6), we have

$$\begin{array}{cc} \frac{1}{\phi^{2}}\frac{d\phi }{dS} & =-\beta +\frac{\gamma }{S}. \end{array}$$

By solving this first-order differential equation and then using (7), we get

$$\begin{array}{c} \frac{d}{dt}\;\text{ln}\;S=\frac{1}{\phi }=\beta \left(S-S_{0}-I_{0}\right)-\gamma \;\text{ln}\;(\frac{S}{S_{0}}) \end{array}$$ (8)

which is equivalent to

$$\begin{array}{c} \frac{1}{\beta \left(S-S_{0}-I_{0}\right)-\gamma \;\text{ln}\;(\frac{S}{S_{0}})}d\;\text{ln}\;S=dt. \end{array}$$ (9)

The exact solution of (9) is called the *analytical solution*. By using power series approximation of ln(*S*/*S*_0_), we obtain an approximate solution called a *semi-analytical solution*. Since we assumed that *S*_0_ + *I*_0_ = 1 and the term $\;\text{ln}\;(\frac{S}{S_{0}})$ can be approximated by $\frac{S}{S_{0}}-1$, it follows that (9) becomes

$$\begin{array}{c} \frac{1}{e^{\;\text{ln}\;S}\left(\beta -\frac{\gamma }{S_{0}}\right)+\gamma -\beta }d\;\text{ln}\;S=dt. \end{array}$$ (10)

We note here that the assumption *I*_0_ > 0 implies *S*_0_ < 1. It follows that the terms *βS*_0_ − *γ* and *γ* − *β* cannot be zero at the same time. Now, we consider two cases.

Case 1: *β* = *γ*. Integrating both sides of 10 gives

$$\begin{array}{c} S\left(t\right)=\frac{S_{0}}{1+\beta (1-S_{0})t}. \end{array}$$ (11)

Case 2: *β* ≠ *γ* By integrating both sides of 10, we simply get

$$\begin{array}{c} S\left(t\right)=\frac{\left(\beta -\gamma \right)S_{0}}{\beta \left(1-S_{0}\right)e^{(\beta -\gamma )t}+\beta S_{0}-\gamma }. \end{array}$$ (12)

Once we obtain the approximate solution *S*(*t*), we can derive the corresponding solutions of *I*(*t*) (using (4) and (8)) and *R*(*t*) = 1 − *S*(*t*) − *I*(*t*) as follows:

$$\begin{array}{c} I(t)=-S\left(t\right)+1+\frac{\gamma }{\beta }\;\text{ln}\;(\frac{S(t)}{S_{0}}), \end{array}$$ (13)

$$\begin{array}{c} R(t)=\frac{\gamma }{\beta }\;\text{ln}\;(\frac{S_{0}}{S(t)}). \end{array}$$ (14)

The set of (*S*(*t*), *I*(*t*), *R*(*t*)) for *t* ≥ 0 presented in (12)-(14) is called a semi-analytical solution.

### Dirichlet series solutions of the SIR model

Let *u*(*t*) = ln(*S*(*t*)/*S*_0_). Then, (8) is equivalent to

$$\begin{array}{c} u^{\prime }+\gamma u+\beta =\beta S_{0}e^{u}. \end{array}$$ (15)

Since this first-order differential equation has no exact non-parametric analytical solution, we consider its Dirichlet series solution. We first set up some notations for ease of computation. Considering 0 = λ_0_ < λ_1_ < … and $a_{i}\in C\backslash \left\{0\right\}$, let

$$\begin{array}{cc} u(t) & =\sum_{i=0}^{\infty }a_{i}e^{-\lambda_{i}t}\\ & =\sum_{i=0}^{n-1}a_{i}e^{-\lambda_{i}t}+\sum_{i=n}^{\infty }a_{i}e^{-\lambda_{i}t}\\ & =:u_{1,n}\left(t\right)+u_{2,n}\left(t\right), \end{array}$$ (16)

and let

$$\begin{array}{c} F\left(u,u^{\prime }\right)=u^{\prime }+\gamma u-\beta S_{0}e^{u}+\beta . \end{array}$$ (17)

Then (15) is equivalent to *F*(*u*, *u*′) = 0. By Taylor series expansion we have,

$$\begin{array}{c} 0=F\left(u,u^{\prime }\right)=F\left(u_{1,n}+u_{2,n},u_{1,n}^{\prime }+u_{2,n}^{\prime }\right)=F\left(u_{1,n},u_{1,n}^{\prime }\right)+A_{n}, \end{array}$$ (18)

where

$$\begin{array}{cc} A_{n} & =u_{2,n}\left(\gamma -\beta S_{0}e^{u_{1,n}}\right)+u_{2,n}^{\prime }-\frac{{\left(u_{2,n}\right)}^{2}\beta S_{0}e^{u_{1,n}}}{2}+\cdots \\ & =a_{n}e^{-\lambda_{n}t}\left(\gamma -\beta S_{0}e^{a_{0}}-\lambda_{n}\right)+\text{terms}\;\text{with}\;\text{higher}\;\text{exponents.} \end{array}$$ (19)

The following result provides properties of the Dirichlet series solution of (15) which is equivalent to (18).

**Theorem 1** *Let* $\;\text{ln}\;\left(S\left(t\right)/S_{0}\right)=u\left(t\right)=\sum_{i=0}^{\infty }a_{i}e^{-\lambda_{i}t}$ , 0 = λ_0_ < λ_1_ < …, $a_{i}\in C\backslash \left\{0\right\}$ *be a Dirichlet series satisfying* (15). *Then*,

- 1 − *S*_∞_ + (*γ*/*β*)ln(*S*_∞_/*S*_0_) = 0,
- λ_1_ = *γ* − *βS*_∞_,
- *a*_0_ = ln(*S*_∞_/*S*_0_) *and* $a_{1}=-a_{0}-\sum_{i=2}^{\infty }a_{i}$,
- for *n* ≥ 2 if $0\neq l_{n}=\sum_{j=2}^{n}\sum_{\begin{array}{c} i_{1}+\cdots +i_{j}=n\\ i_{1},\ldots ,i_{j}>0 \end{array}}a_{i_{1}}\cdots a_{i_{j}}/j!,$ then λ_*n*_ = *nλ*_1_ and $a_{n}=-\frac{\beta S_{\infty }l_{n}}{\left(n-1\right)\lambda_{1}}$.

**Proof.** It is obvious that *a*_0_ = ln(*S*_∞_/*S*_0_) and $u\left(0\right)=0=\sum_{i=0}^{\infty }a_{i}$. Consider (18) with *n* = 1, we see that $F\left(u_{1,1},u_{1,1}^{\prime }\right)=\gamma a_{0}-\beta S_{0}e^{a_{0}}+\beta$ is a constant and the first term of *A*_1_ is non-constant, $a_{1}e^{-\lambda_{1}t}\left(\gamma -\beta S_{0}e^{a_{0}}-\lambda_{1}\right)$. This implies that $0=\gamma a_{0}-\beta S_{0}e^{a_{0}}+\beta$ and $\lambda_{1}=\gamma -\beta S_{0}e^{a_{0}}=\gamma -\beta S_{\infty }$. The former is equivalent to

$$\begin{array}{c} 1-S_{\infty }+\left(\gamma /\beta \right)\;\text{ln}\;\left(S_{\infty }/S_{0}\right)=0. \end{array}$$ (20)

Consider (18) with *n* = 2, observe that the first term (least exponent) of *A*_2_ is $a_{2}e^{-\lambda_{2}t}\left(\gamma -\beta S_{0}e^{a_{0}}-\lambda_{2}\right)\neq 0$ since *a*_2_ ≠ 0 and λ_1_ < λ_2_. That means that this term would be canceled by a term in $F(u_{1,2},u_{1,2}^{\prime })$. Note that the exponents of the terms in $F(u_{1,2},u_{1,2}^{\prime })$ are linear combination of λ_1_. This implies that λ_2_ = *nλ*_1_ for some integer *n* ≥ 2. Since $F(u_{1,2},u_{1,2}^{\prime })$ has the term $-\beta S_{\infty }\frac{a_{1}^{2}}{2}e^{-2\lambda_{1}t}=-\beta S_{\infty }l_{2}e^{-2\lambda_{1}t}\neq 0$, it follows that λ_2_ = 2λ_1_ and then the sum of coefficients of the terms $e^{-\lambda_{2}t}$ and $e^{-2\lambda_{1}t}$ must be zero, i.e., $a_{2}=-\frac{\beta S_{\infty }l_{2}}{\lambda_{1}}$. Now consider (18) with a fixed *n* > 2 and assume that λ_*k*_ = *k*λ_1_ for 1 ≤ *k* ≤ *n* − 1. The first term of *A*_*n*_ is $a_{n}e^{-\lambda_{n}t}\left(\gamma -\beta S_{0}e^{a_{0}}-\lambda_{n}\right)\neq 0$ since *a*_*n*_ ≠ 0 and λ_1_ < λ_*n*_. This means that the exponent −λ_*n*_*t* must appear in $F(u_{1,n},u_{1,n}^{\prime })$. Note that

$$\begin{array}{cc} F(u_{1,n},u_{1,n}^{\prime }) & =-\sum_{i=0}^{n-1}i\lambda_{1}a_{i}e^{-i\lambda_{1}t}+\gamma \sum_{i=0}^{n-1}a_{i}e^{-i\lambda_{1}t}-\beta S_{\infty }e^{\sum_{i=1}^{n-1}a_{i}e^{-i\lambda_{1}t}}+\beta . \end{array}$$ (21)

Since the exponent −λ_*n*_*t* must be equal to −*j*λ_1_*t* for some *j* ≥ *n*, it implies that the term with the exponent −λ_*n*_*t* can be extracted from the term $-\beta S_{\infty }e^{\sum_{i=1}^{n-1}a_{i}e^{-i\lambda_{1}t}}$, see (21). By the series expansion, the term $-\beta S_{\infty }e^{\sum_{i=1}^{n-1}a_{i}e^{-i\lambda_{1}t}}$ can be written as

$$\begin{array}{c} -\beta S_{\infty }(1+\sum_{i=1}^{n-1}a_{i}e^{-i\lambda_{1}t}+\frac{{\left(\sum_{i=1}^{n-1}a_{i}e^{-i\lambda_{1}t}\right)}^{2}}{2!}+\frac{{\left(\sum_{i=1}^{n-1}a_{i}e^{-i\lambda_{1}t}\right)}^{3}}{3!}+\cdots ) \end{array}$$

which contains the term of exponent *nλ*_1_ in the form

$$\begin{array}{c} -\beta S_{\infty }(\sum_{j=2}^{n}\sum_{i_{1}+\cdots +i_{j}=ni_{1},\ldots ,i_{j}>0}a_{i_{1}}\cdots a_{i_{j}}/j!)e^{-n\lambda_{1}t}=-\beta S_{\infty }l_{n}e^{-n\lambda_{1}t}\neq 0. \end{array}$$

Hence, λ_*n*_ = *nλ*_1_ and also $a_{n}=-\frac{\beta S_{\infty }l_{n}}{\left(n-1\right)\lambda_{1}}$. We have done the proof.

Note that Eqs (13), (14) and (20) imply that

$$I_{\infty }=\underset{t\to \infty }{\text{lim}}I\left(t\right)=0\;\;\text{and}\;\;R_{\infty }=\underset{t\to \infty }{\text{lim}}R\left(t\right)=1-S_{\infty }.$$

This means that the solution set of the SIR model,

$$\{\left(S\left(t\right),I\left(t\right),R\left(t\right)\right)|S\left(0\right)=S_{0},I\left(0\right)=I_{0},R\left(0\right)=0\},$$

approaches (*S*_∞_, 0, 1 − *S*_∞_). Consider a Dirichlet series approximant which is an approximate form of the solution *u*(*t*), say

$$u_{N}\left(t\right)=\sum_{i=0}^{N}a_{i}e^{-\lambda_{i}t}$$

for some N∈N. By the previous theorem, to obtain *a*_*n*_ we need to get *l*_*n*_ which depends on *a*_1_, …, *a*_*n*−1_ and $a_{1}=a_{0}-\sum_{i=2}^{n}a_{i}$. It implies that *l*_*i*_ for each *i* = 3, …, *N* may not be unique and the values of *a*_1_, …, *a*_*n*_ can be non-unique complex numbers. This provides many approximate forms of the complex Dirichlet series *u*(*t*) which are not applicable to computer programming. We need the series *u*(*t*) to be a series of real coefficients. The results in Theorem 1 induce us to consider a specific Dirichlet series solution whose exponents satisfy λ_*n*_ = *nλ*_1_ for n∈N. The next results are more applicable for achieving the real Dirichlet series *u*(*t*).

**Lemma 2** *If* $f\left(t\right)=e^{\sum_{i=1}^{\infty }a_{i}e^{-\lambda_{i}t}}$ *and* $\sum_{i=0}^{\infty }a_{i}=0$ , *then the* *n*^*th*^ *derivative of* *f* *with respect to* *t at t* = 0 *satisfies*

$$f^{\left(n\right)}\left(0\right)={\left(-1\right)}^{n}e^{-a_{0}}\sum_{k=1}^{n}\left({}_{k-1}^{n-1}\right)c_{n-k}b_{k},$$

where $c_{k}=\left(\begin{array}{c} k-1\\ 0 \end{array}\right)c_{k-1}b_{1}+\left(\begin{array}{c} k-1\\ 1 \end{array}\right)c_{k-2}b_{2}+\cdots +\left(\begin{array}{c} k-1\\ k-1 \end{array}\right)c_{0}b_{k}$ for *k* ≥ 1, *c*_0_ = 1 and $b_{k}=\sum_{i=1}^{\infty }a_{i}\lambda_{i}^{k}$.

The proof simply follows from the general Leibniz rule and the mathematical induction.

**Theorem 3** *Let* $\;\text{ln}\;\left(S\left(t\right)/S_{0}\right)=u\left(t\right)=\sum_{i=0}^{\infty }a_{i}e^{-\lambda_{i}t}$ , 0 = λ_0_ < λ_1_ < …, $a_{i}\in R\backslash \left\{0\right\}$ *be a Dirichlet series satisfying* (15). *Let* $b_{n}=\sum_{i=1}^{\infty }a_{i}\lambda_{i}^{n}$ *for* *n* ≥ 0. *Then*,

$$b_{0}=-a_{0},\;b_{1}=\beta I_{0},\;b_{n}=\gamma b_{n-1}-\beta S_{0}\sum_{i=1}^{n-1}\left({}_{i-1}^{n-2}\right)c_{n-1-i}b_{i}$$

*for* *n* = 2, …, *where* $c_{k}=\left(\begin{array}{c} k-1\\ 0 \end{array}\right)c_{k-1}b_{1}+\cdots +\left(\begin{array}{c} k-1\\ k-1 \end{array}\right)c_{0}b_{k}$ for *k* ≥ 1 *and* *c*_0_ = 1.

**Proof.** By the result in Theorem 1, we have *b*_0_ = −*a*_0_. By plugging the Dirichlet series *u*(*t*) into (15), we get

$$\begin{array}{c} \beta +\gamma a_{0}+\sum_{i=1}^{\infty }a_{i}\left(\gamma -\lambda_{i}\right)e^{-\lambda_{i}t}=\beta S_{\infty }e^{\sum_{i=1}^{\infty }a_{i}e^{-\lambda_{i}t}}. \end{array}$$ (22)

Eq (22) at *t* = 0 implies that *b*_1_ = *βI*_0_. By taking the (*n* − 1)^*th*^ derivative both sides of (22) with respect to *t* and then plugging *t* = 0 together with the result of Lemma 2, we obtain that

$$\begin{array}{cc} & \sum_{i=1}^{\infty }{\left(-1\right)}^{n-1}a_{i}\lambda_{i}^{n-1}\left(\gamma -\lambda_{i}\right)\\ & =\beta S_{\infty }{\left(-1\right)}^{n-1}e^{-a_{0}}\sum_{i=1}^{n-1}n-2i-1c_{n-1-i}b_{i}. \end{array}$$

This implies the result of this theorem.

### Dirichlet series approximants of the basic SIR model

Followed by Theorem 1, it is intuitive to consider the case of λ_*i*_ = *i*λ_1_. This implies by setting in Theorem 3 that

$$\begin{array}{cc} \sum_{i=1}^{\infty }a_{i} & =-a_{0},\\ \sum_{i=1}^{\infty }ia_{i} & =b_{1}/\lambda_{1},\\ & \vdots \\ \sum_{i=1}^{\infty }i^{n}a_{i} & =b_{n}/\lambda_{1}^{n},\\ & \vdots \end{array}$$

which can be written in the matrix form as follows:

$$[\begin{array}{ccccc} 1 & 1 & \cdots & 1 & \cdots \\ 1 & 2 & \cdots & n & \cdots \\ 1^{2} & 2^{2} & \cdots & n^{2} & \cdots \\ \vdots & \vdots & \vdots & \vdots & \cdots \\ 1^{n} & 2^{n} & \cdots & n^{n} & \cdots \\ \vdots & \vdots & \vdots & \vdots & \vdots \end{array}][\begin{array}{c} a_{1}\\ a_{2}\\ a_{3}\\ \vdots \\ a_{n}\\ \vdots \end{array}]=[\begin{array}{c} -a_{0}\\ b_{1}/\lambda_{1}\\ b_{2}/\lambda_{1}^{2}\\ \vdots \\ b_{n}/\lambda_{1}^{n}\\ \vdots \end{array}],$$

where *a*_0_ = ln(*S*_∞_/*S*_0_), *S*_∞_ can be derived by (20), and *b*_1_, *b*_2_, … can be obtained by the result of Theorem 3.

Now consider an approximate form of the Dirichlet series $u_{N}\left(t\right)=\sum_{i=0}^{N}a_{i}e^{-i\lambda_{1}t}$ satisfying (15). Then by the previous results, we get

$$\begin{array}{cc} a_{0} & =\;\text{ln}\;(S_{\infty }/S_{0}),\\ 1 & =S_{\infty }-\frac{\gamma }{\beta }\;\text{ln}\;(\frac{S_{\infty }}{S_{0}}),\\ \lambda_{1} & =\gamma -\beta S_{\infty }, \end{array}$$

and *a*_1_, …, *a*_*N*_ can be achieved by solving

$$\begin{array}{c} {}[\begin{array}{cccc} 1 & 1 & \cdots & 1\\ 1 & 2 & \cdots & N\\ 1^{2} & 2^{2} & \cdots & N^{2}\\ \vdots & \vdots & \vdots & \vdots \\ 1^{N-1} & 2^{N-1} & \cdots & N^{N-1} \end{array}][\begin{array}{c} a_{1}\\ a_{2}\\ a_{3}\\ \vdots \\ a_{N} \end{array}]=[\begin{array}{c} -a_{0}\\ b_{1}/\lambda_{1}\\ b_{2}/\lambda_{1}^{2}\\ \vdots \\ b_{N}/\lambda_{1}^{N-1} \end{array}]. \end{array}$$ (23)

The existence of the solution *a*_1_, *a*_2_, …, *a*_*N*_ is due to the existence of the inverse of the Vandermonde matrix [28]. Note here that this matrix equation is solvable when *N*, *a*_0_, λ_1_, *b*_1_, …, *b*_*N*_ are known. After *u*_*N*_(*t*) is obtained by solving the matrix equation above, an approximation of the solution *S*(*t*), say *S*_*N*_(*t*), is derived by

$$\begin{array}{c} S_{N}\left(t\right)=S_{0}e^{u_{N}\left(t\right)}. \end{array}$$ (24)

The other approximations of the solutions *I*(*t*) and *R*(*t*), say *I*_*N*_(*t*) and *R*_*N*_(*t*) respectively, are as follows:

$$\begin{array}{c} I_{N}\left(t\right)=-S_{N}\left(t\right)+1+\frac{\gamma }{\beta }\;\text{ln}\;(\frac{S_{N}\left(t\right)}{S_{0}}) \end{array}$$ (25)

$$\begin{array}{c} R_{N}\left(t\right)=\frac{\gamma }{\beta }\;\text{ln}\;(\frac{S_{0}}{S_{N}\left(t\right)}). \end{array}$$ (26)

It is implied by Theorem 3 that *c*_*k*_ can be written in terms of *b*_1_, …, *b*_*k*_. The process to get *c*_*k*_ presented in Theorem 3 is so applicable that any mathematical programming can perform the task. The terms *b*_*k*_ can be derived after the terms *c*_0_, …, *c*_*k*−2_ were known.

### Numerical simulations for the SIR model

In this subsection, we present an algorithm to obtain the Dirichlet series approximants *S*_*N*_(*t*), *I*_*N*_(*t*), *R*_*N*_(*t*). According to the previous results, the process of achieving the approximant of the SIR model is as follows:

Step 1: Setting *c*_0_ = 1, then *c*_*k*_, k∈N, can be written in terms of *b*_1_, *b*_2_, …, *b*_*k*_ by using $c_{k}=\left(\begin{array}{c} k-1\\ 0 \end{array}\right)c_{k-1}b_{1}+\left(\begin{array}{c} k-1\\ 1 \end{array}\right)c_{k-2}b_{2}+\cdots +\left(\begin{array}{c} k-1\\ k-1 \end{array}\right)c_{0}b_{k}$.

Step 2: Solving 1 − *S*_∞_ + (*γ*/*β*)ln(*S*_∞_/*S*_0_) = 0 for *S*_∞_, setting *a*_0_ = ln(*S*_∞_/*S*_0_), λ_1_ = *r* − *βS*_∞_ and *b*_0_ = −*a*_0_.

Step 3: Setting *b*_1_ = *βI*_0_, then *b*_*k*_ can be written in terms of *b*_1_, …, *b*_*k*−1_ by using Steps 1 and $b_{k}=\gamma b_{k-1}-\beta S_{0}\sum_{i=1}^{k-1}\left({}_{i-1}^{k-2}\right)c_{k-1-i}b_{i}$. This step gives the values of *b*_1_, *b*_2_, …, *b*_*N*_.

Step 4: Solving the matrix Eq (23) for *a*_1_, *a*_2_, …, *a*_*N*_. In this step we get $u_{N}\left(t\right)=\sum_{i=0}^{N}a_{i}e^{-i\lambda_{1}t}$.

Step 5: Plotting *S*_*N*_(*t*), *I*_*N*_(*t*), *R*_*N*_(*t*) by using Eqs (24–26)

We first show manually how to get some *c*_*n*_ by performing Step 1

$$\begin{array}{cc} c_{0} & =1,\\ c_{1} & =b_{1},\\ c_{2} & =c_{1}b_{1}+b_{2}=b_{1}^{2}+b_{2},\\ c_{3} & =b_{1}^{3}+3b_{1}b_{2}+b_{3},\\ c_{4} & =\left(b_{1}^{3}+3b_{1}b_{2}+b_{3}\right)b_{3}+3\left(b_{1}^{2}+b_{2}\right)b_{2}+3b_{1}b_{3}+b_{4}, \end{array}$$

and then we manually derive *b*_*n*_ by implementing Step 3 and the result in Step 1

$$\begin{array}{cc} b_{0} & =-a_{0}=-\;\text{ln}\;\left(S_{\infty }/S_{0}\right),\\ b_{1} & =\beta I_{0},\\ b_{2} & =\left(\gamma -\beta S_{0}\right)b_{1}=\left(\gamma -\beta S_{0}\right)\beta I_{0},\\ b_{3} & =\gamma \beta I_{0}\left(\gamma -\beta S_{0}\right)-\beta^{2}S_{0}I_{0}\left(\gamma +\beta I_{0}-\beta S_{0}\right),\\ b_{4} & =\beta I_{0}\left(\gamma -\beta S_{0}\right)\left(\gamma^{2}-2\gamma \beta S_{0}-3\beta^{2}I_{0}S_{0}+\beta^{2}S_{0}^{2}\right)-\gamma \beta^{3}I_{0}^{2}S_{0}-\beta^{4}I_{0}^{3}S_{0}+\beta^{4}S_{0}^{2}I_{0}^{2}. \end{array}$$

**Fig 1:**
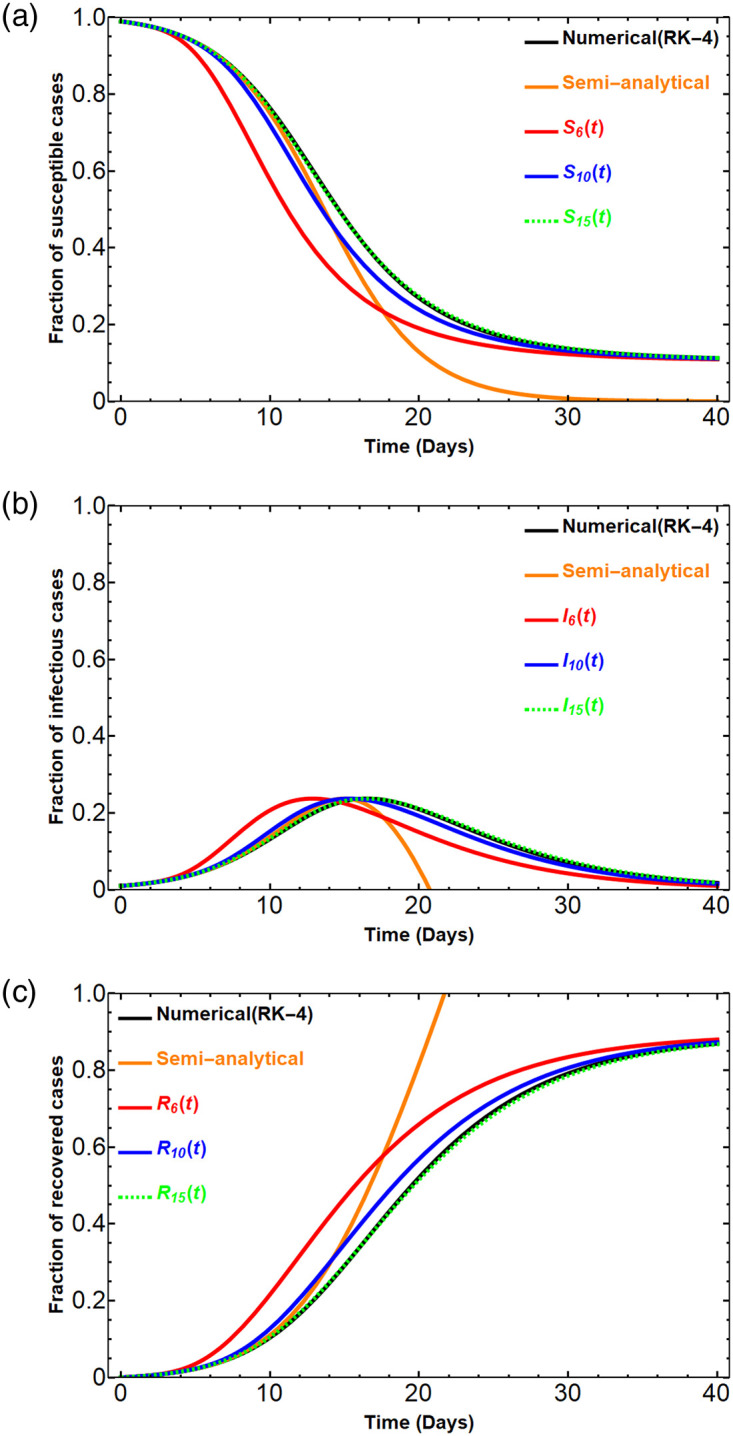
The solution graph comparison. Under the assumed parameters that *β* = 0.5, *γ* = 0.2 and the initial conditions *S*_0_ = 0.99, *I*_0_ = 0.01, *R*_0_ = 0, the three figures show the graphs of the proportions of susceptible, infectious, and recovered cases. The figures show the comparison of the approximate solution obtained by the numerical method (RK-4), the Dirichlet series approximants *S*_*N*_(*t*), *I*_*N*_(*t*), *R*_*N*_(*t*), for *N* = 6, 10, 15, and the approximate analytical using (12).

**Fig 2:**
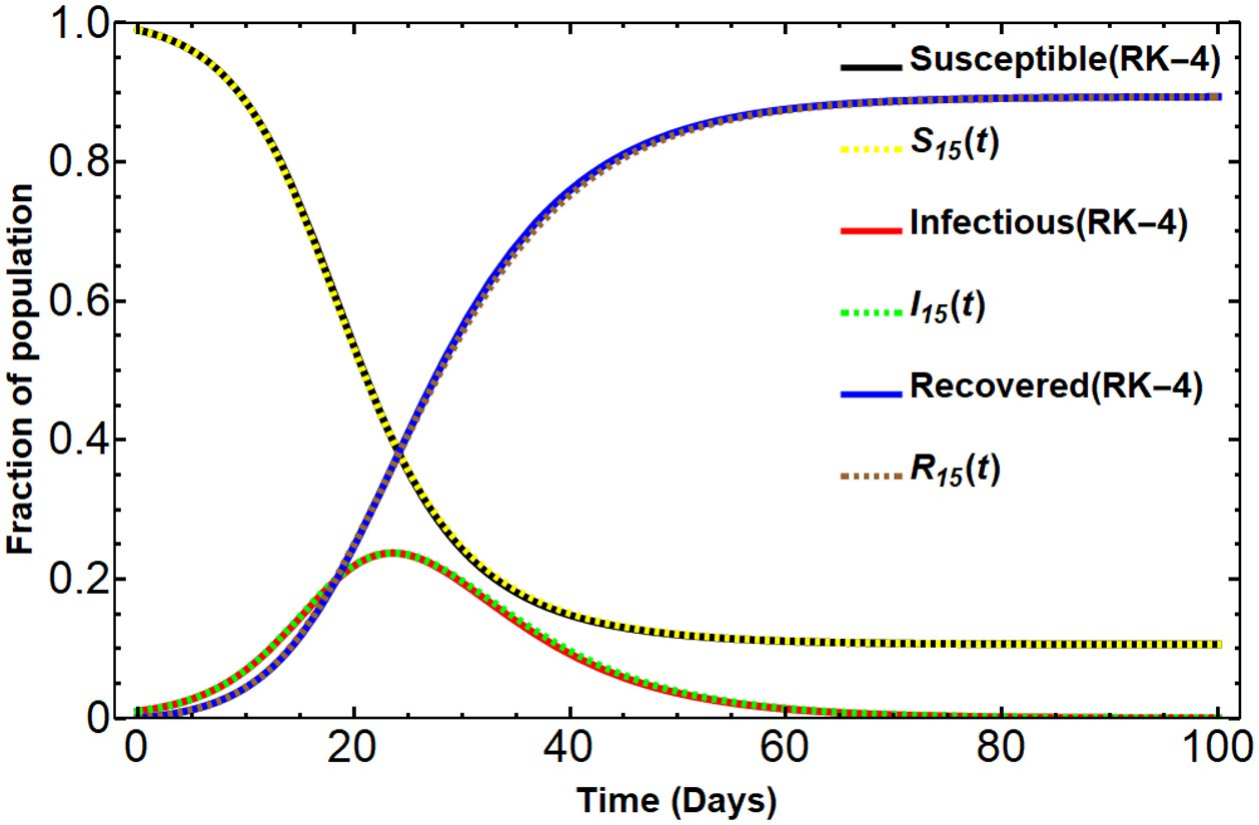
The Dirichlet approximations order 15 Vs RK-4 method. Under the assumed parameters related to the COVID-19 situation [2] *β* = 0.35, *γ* = 0.14, and the initial conditions *S*_0_ = 0.99, *I*_0_ = 0.01, *R*_0_ = 0, the figure compares the graphs of the SIR model solutions obtained by the numerical method (RK-4) in the solid lines and the Dirichlet series approximants *S*_15_(*t*), *I*_15_(*t*), *R*_15_(*t*) in the dashed lines.

Fig 1 shows the comparison of the SIR solutions obtained by RK-4 method, Dirichlet series approximants (*S*_6_(*t*), *S*_10_(*t*), *S*_15_(*t*)), and the semi-analytical solution. Even though the graph of the semi-analytical solution using (12) is close to the numerical solution at the beginning time interval, it has no biological meaning after 21 days passed. The infectious proportion is negative and the recovered fraction is higher than 1 when 21 days have passed, see Fig 1. The approximants *S*_*N*_(*t*), *I*_*N*_(*t*), *R*_*N*_(*t*) are close to the numerical solution on the beginning and ending time intervals. As shown in Fig 1, the better approximation comes with the larger *N*. Figs 1 and 2 also illustrate that a small positive integer *N* is enough to achieve a suitable numerical approximation of the SIR solution.

Here we show *u*_15_(*t*) corresponding to the given parameters and initial conditions in Fig 2.

$$\begin{array}{cc} & u_{15}\left(t\right)=-2.23526+0.667927e^{-1.54406t}-10.1641e^{-1.44112t}+72.3135e^{-1.33818t}\\ & -319.195e^{-1.23524t}+978.052e^{-1.13231t}-2205.03e^{-1.02937t}+3781.85e^{-0.926433t}\\ & -5030.29e^{-0.823496t}+5240.03e^{-0.720559t}-4284.69e^{-0.617622t}+2736.93e^{-0.514685t}\\ & -1348.28e^{-0.411748t}+500.112e^{-0.308811t}-133.847e^{-0.205874t}+23.7809e^{-0.102937t} \end{array}$$

By using this real Dirichlet series *u*_15_(*t*) with (24)-(26), we get the approximated solution of the SIR model. The results above also give the long-run behavior of the considered SIR model in Fig 2 as follows:

$$\left(S_{\infty },I_{\infty },R_{\infty }\right)=\left(S_{\infty },0,1-S_{\infty }\right)=\left(0.105894,0,0.894106\right).$$

As seen in Figs 1 and 2, the approximations *S*_15_(*t*), *I*_15_(*t*), *R*_15_(*t*) are perfectly matched to the numerical solution obtained by the RK-4 method.

We note here that the approximate closed-form derived by our method presents an approximate long-run behavior of the system while the iterative RK-4 method and the exact parametric solution in [19] do not present it.

### Error comparison of the SIR model

**Fig 3:**
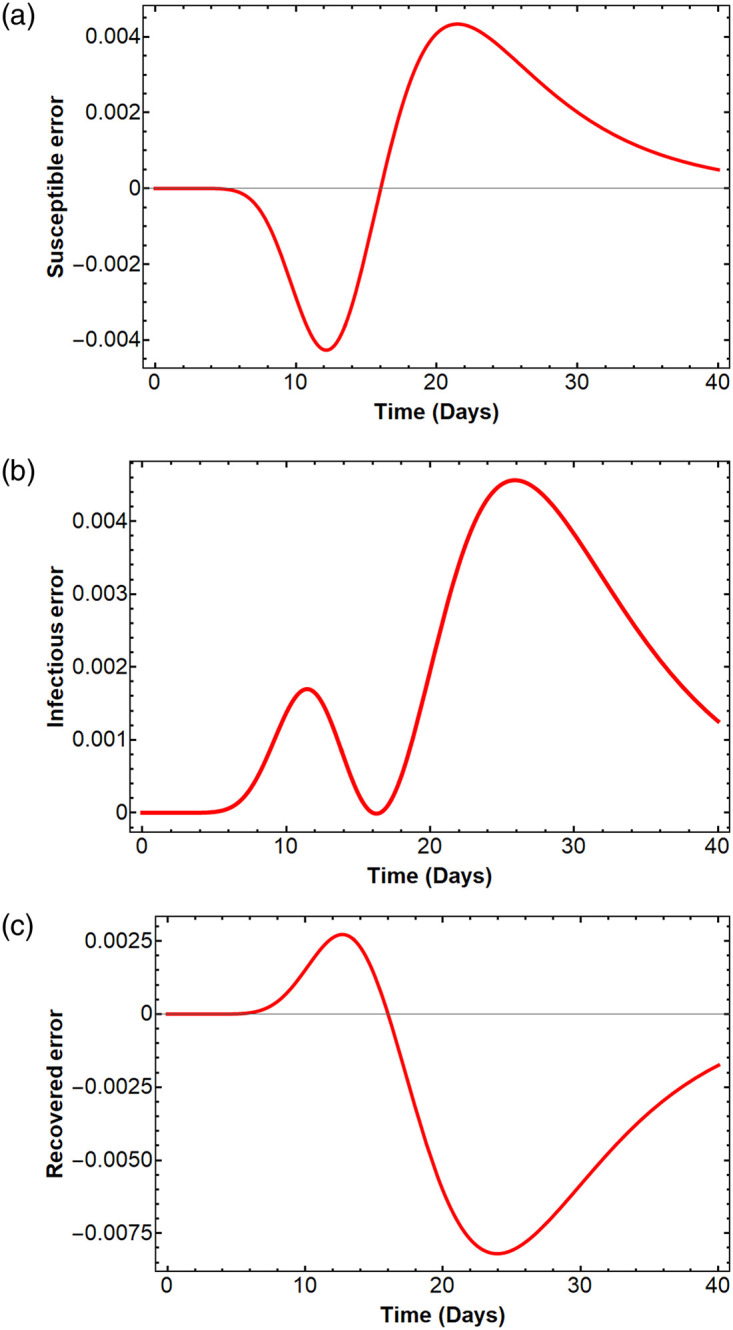
Error of the solutions *S*_15_(*t*), *I*_15_(*t*), *R*_15_(*t*) compared with the solutions obtained from the RK-4 method.

Since the exact values of the solutions *S*(*t*), *I*(*t*), and *R*(*t*) at any time *t* are unknown because the model has no closed-form solution, we illustrate the error of our method comparing with the RK-4 method. Fig 3 shows the errors of *S*_15_(*t*), *I*_15_(*t*), *R*_15_(*t*) compared with the RK-4 method. The graphs illustrate the gaps between the solutions from the RK-4 method and our method; i.e., the graphs show the differences *S*_15_(*t*) − *S*(*t*), *I*_15_(*t*) − *I*(*t*), and *R*_15_(*t*) − *R*(*t*) at any time *t*. The mean squared errors of *S*_15_, *I*_15_(*t*), *R*_15_(*t*) compared with the numerical solutions *S*(*t*), *I*(*t*), *R*(*t*) are 5.88606 × 10^−6^, 5.81967 × 10^−6^ and 0.0000187405, respectively.

## A special Dirichlet series solutions of the SEIR model

In this section, we consider another compartment model consisting of the susceptible population *S*, the exposed population *E*, the infectious population *I*, and the recovered population *R* called the SEIR model whose system of differential equations is as follows:

$$\begin{array}{c} \frac{dS}{dt}=-\beta IS, \end{array}$$ (27)

$$\begin{array}{c} \frac{dE}{dt}=\beta IS-\sigma E, \end{array}$$ (28)

$$\begin{array}{c} \frac{dI}{dt}=\sigma E-\gamma I, \end{array}$$ (29)

$$\begin{array}{c} \frac{dR}{dt}=\gamma I, \end{array}$$ (30)

where the initial conditions are assumed to be *S*(0) = *S*_0_ > 0, *E*(0) = *E*_0_ > 0, *I*(0) = *I*_0_ > 0, *R*(0) = 0, and $\beta ,\sigma ,\gamma \in R_{0}^{+}$. Additionally, the total proportion at any time *t* is assumed to satisfy *S*(*t*) + *E*(*t*) + *I*(*t*) + *R*(*t*) = 1.

Let us say that we want an approximated solution *R*(*t*) of this model to be a specific Dirichlet series in the form:

$$\begin{array}{c} R\left(t\right)=\sum_{i=0}^{\infty }h_{i}e^{-i\lambda_{1}t},\;\;\;h_{i},\lambda_{1}\in R\backslash \left\{0\right\}. \end{array}$$ (31)

By plugging (31) in (30) and (29), it is easy to see that the solutions *I*(*t*) and *E*(*t*) can be written in a similar Dirichlet form of *R*(*t*) and the constant terms of the Dirichlet expansion of *I*(*t*) and *E*(*t*) are zero. This implies that

$$\begin{array}{c} I_{\infty }:=\underset{t\to \infty }{\text{lim}}I\left(t\right)=0\;\;\text{and}\;\;E_{\infty }:=\underset{t\to \infty }{\text{lim}}E\left(t\right)=0. \end{array}$$

We also simply obtain

$$\begin{array}{c} S_{\infty }+R_{\infty }:=\underset{t\to \infty }{\text{lim}}S\left(t\right)+\underset{t\to \infty }{\text{lim}}R\left(t\right)=1. \end{array}$$ (32)

By substituting (30) in (27), we get

dS=-βSdR.

Solving this separable differential equation leads to

$$\begin{array}{c} \;\text{ln}\;(\frac{S(t)}{S_{0}})=-\frac{\beta }{\gamma }R\left(t\right). \end{array}$$ (33)

By (32) and (33), we have

$$\begin{array}{c} h_{0}=R_{\infty }=-\frac{\gamma }{\beta }\;\text{ln}\;(\frac{1-R_{\infty }}{S_{0}}). \end{array}$$ (34)

Eq (31) with *t* = 0 implies that

$$\sum_{i=1}^{\infty }h_{i}=-h_{0}.$$

Plugging (31) in (30) and setting *t* = 0 lead to

$$\sum_{i=1}^{\infty }ih_{i}=-\frac{\gamma I_{0}}{\lambda_{1}}.$$

Now setting *D*_*n*_ ≔ *γ*(*σE*^(*n*)^(0) − *γI*^(*n*)^(0)), where *E*^(*n*)^(0) and *I*^(*n*)^(0) are the n^th^ derivative of *E*(*t*) and *I*(*t*) at *t* = 0, respectively, for n∈N∪{0}. Taking the first derivative of (30) with respect to *t* and then plugging *t* = 0 lead us to

$$\sum_{i=1}^{\infty }i^{2}h_{i}=\frac{\gamma (\sigma E_{0}-\gamma I_{0})}{\lambda_{1}^{2}}=\frac{D_{0}}{\lambda_{1}^{2}}.$$

If we keep taking derivatives till the n^th^ derivative of *R*(*t*) with respect to *t* and plugging *t* = 0, we should get

$$\sum_{i=1}^{\infty }i^{n}h_{i}={\left(-1\right)}^{n}\frac{\gamma (\sigma E^{\left(n-2\right)}\left(0\right)-\gamma I^{\left(n-2\right)}\left(0\right))}{\lambda_{1}^{n}}={\left(-1\right)}^{n}\frac{D_{n-2}}{\lambda_{1}^{n}}.$$

An approximated solution *R*(*t*) could be considered in the form

$$R_{N}\left(t\right)=\sum_{i=0}^{N}h_{i}e^{-i\lambda_{1}t},$$

where *h*_0_ is obtained by (34) and other *h*_*i*_ are achieved by solving the matrix equation:

$$\begin{array}{c} {}[\begin{array}{cccc} 1 & 1 & \cdots & 1\\ 1 & 2 & \cdots & N\\ 1^{2} & 2^{2} & \cdots & N^{2}\\ \vdots & \vdots & \vdots & \vdots \\ 1^{N-1} & 2^{N-1} & \cdots & N^{N-1} \end{array}][\begin{array}{c} h_{1}\\ h_{2}\\ h_{3}\\ \vdots \\ h_{N} \end{array}]=[\begin{array}{c} -h_{0}\\ -\gamma I_{0}/\lambda_{1}\\ D_{0}/\lambda_{1}^{2}\\ \vdots \\ {\left(-1\right)}^{N-1}D_{N-3}/\lambda_{1}^{N-1} \end{array}]. \end{array}$$ (35)

Note that *D*_*n*_ can be recursively solved by

$$\begin{array}{c} D_{n}=\gamma (\sigma E^{\left(n\right)}\left(0\right)-\gamma I^{\left(n\right)}\left(0\right)), \end{array}$$ (36)

$$\begin{array}{c} I^{\left(n\right)}\left(0\right)=\sigma E^{\left(n-1\right)}\left(0\right)-\gamma I^{\left(n-1\right)}\left(0\right), \end{array}$$ (37)

$$\begin{array}{c} E^{\left(n\right)}\left(0\right)=\beta {\left(IS\right)}^{\left(n-1\right)}\left(0\right)-\sigma E^{\left(n-1\right)}\left(0\right), \end{array}$$ (38)

$$\begin{array}{c} S^{\left(n\right)}\left(0\right)=-\beta {\left(IS\right)}^{\left(n-1\right)}\left(0\right), \end{array}$$ (39)

$$\begin{array}{c} {\left(IS\right)}^{\left(n-1\right)}\left(0\right)=\sum_{k=0}^{n-1}(\begin{array}{c} n-1\\ k \end{array})I^{\left(n-1-k\right)}\left(0\right)S^{\left(k\right)}\left(0\right). \end{array}$$ (40)

Eqs (35)-(40) give us an approximated solution *R*_*N*_(*t*, λ_1_). After the solution

$$R_{N}\left(t,\lambda_{1}\right)=\sum_{i=0}^{N}h_{i}e^{-i\lambda_{1}t}$$

is derived, other solutions can be achieved as follows:

$$\begin{array}{c} I_{N}\left(t,\lambda_{1}\right)=\frac{1}{\gamma }\frac{d}{dt}R_{N}\left(t,\lambda_{1}\right), \end{array}$$ (41)

$$\begin{array}{c} E_{N}\left(t,\lambda_{1}\right)=\frac{1}{\sigma }(\gamma I_{N}\left(t,\lambda_{1}\right)+\frac{d}{dt}I_{N}\left(t,\lambda_{1}\right)), \end{array}$$ (42)

$$\begin{array}{c} S_{N}\left(t,\lambda_{1}\right)=1-E_{N}\left(t,\lambda_{1}\right)-I_{N}\left(t,\lambda_{1}\right)-R_{N}\left(t,\lambda_{1}\right). \end{array}$$ (43)

The approximated solution (*S*_*N*_(*t*), *E*_*N*_(*t*), *I*_*N*_(*t*), *R*_*N*_(*t*)) of the SEIR is obtained by choosing a value of λ_1_.

### Numerical simulations for the SEIR model

In this subsection, we present a method to achieve an approximated solution of the SEIR model by using the results of the previous subsection. Given the system of differential Eqs (27)-(30) with known parameters *β*, *σ*, *γ* and initial conditions *S*_0_, *E*_0_, *I*_0_, *R*_0_, the process to derive the approximated solution is the following:

Step 1: Derive *D*_*n*_ by using (36)-(40).

Step 2: Solving Eq (34) for *h*_0_,

Step 3: Solving the matrix Eq (35) for *h*_1_, *h*_2_, …, *h*_*N*_. In this step we get $R_{N}\left(t,\lambda_{1}\right)=\sum_{i=0}^{N}h_{i}e^{-i\lambda_{1}t}$.

Step 4: Fixed λ_1_ and Plotting *S*_*N*_(*t*), *E*_*N*_(*t*), *I*_*N*_(*t*) and *R*_*N*_(*t*) by using Eqs (41–43).

Note that each *D*_*n*_ is the function of know parameters *β*, *σ*, *γ*, *S*_0_, *E*_0_, *I*_0_. Here are some examples of *D*_*n*_ expressions:

$$\begin{array}{cc} D_{0} & =\gamma (\sigma E_{0}-\gamma I_{0}),\\ D_{1} & =\gamma \sigma \left(\beta I_{0}S_{0}-\sigma E_{0}\right)-\gamma^{2}\left(\sigma E_{0}-\gamma I_{0}\right)\\ D_{2} & =\left(\gamma^{3}+\gamma \sigma \beta S_{0}\right)\left(\sigma E_{0}-\gamma I_{0}\right)-\left(\gamma^{2}\sigma +\gamma \sigma^{2}\right)\left(\beta I_{0}S_{0}-\sigma E_{0}\right)-\gamma \sigma S_{0}{\left(\beta I_{0}\right)}^{2}. \end{array}$$

**Fig 4:**
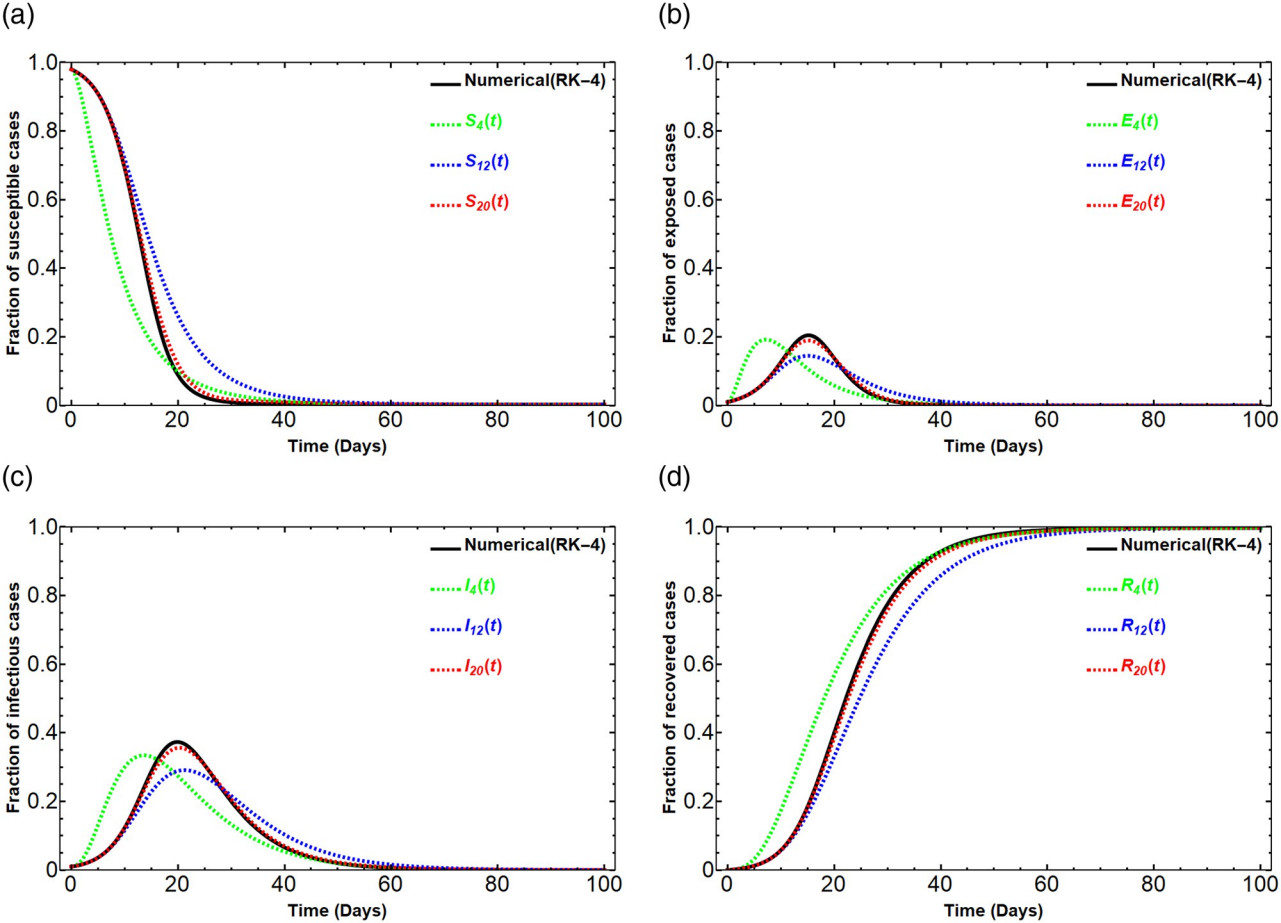
The comparison of solutions *S*_*N*_(*t*), *E*_*N*_(*t*), *I*_*N*_(*t*), *R*_*N*_(*t*) for N = 4, 12, 20 versus the solution obtained by the numerical method RK-4.

**Fig 5:**
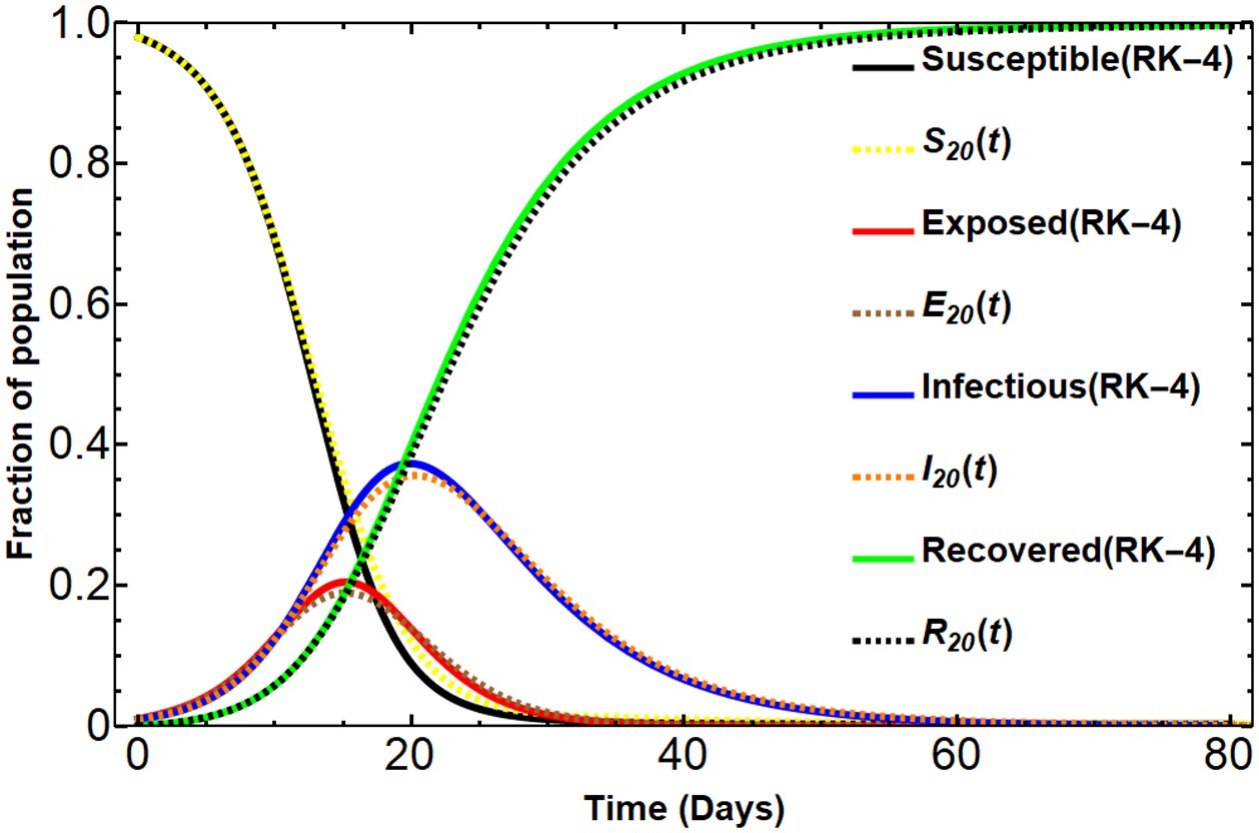
The comparison of the solution (*S*_20_, *E*_20_, *I*_20_, *R*_20_) by our method versus the numerical method RK-4.

To illustrate the results generated by this process, we consider the SEIR model (27)-(30) with the initial conditions *S*_0_ = 0.98, *E*_0_ = 0.01, *I*_0_ = 0.01, and *R*_0_ = 0 under the assumed parameters obtained by [29], *β* = 0.75 per day, *σ* = 1/3 per day, *γ* = 1/8 per day. The comparisons are demonstrated by Figs 4 and 5. Note that in those figures, the value of λ_1_ is chosen to be 0.1. In Fig 4, the graphs of the Dirichlet series approximants *S*_*N*_(*t*), *E*_*N*_(*t*), *I*_*N*_(*t*), and *R*_*N*_(*t*) for *N* = 4, 12, 20 created by our algorithm are compared with the solution from the RK-4 method. As we can see in the figure, the Dirichlet series approximants get closer to the numerical solution obtained by the RK-4 method when *N* gets larger. Fig 5 is shown to conclude that the Dirichlet series approximants order 20, *S*_20_(*t*), *E*_20_(*t*), *I*_20_(*t*) and *R*_20_(*t*) are almost perfectly match the numerical method RK-4. The solution *R*_20_(*t*) obtained by the algorithm is as follows:

$$\begin{array}{c} R_{20}\left(t\right)=0.997535-0.906981e^{-2t}+18.0889e^{-1.9t}-171.28e^{-1.8t}+1023.69e^{-1.7t}\\ -4330.52e^{-1.6t}+13780.6e^{-1.5t}-34219e^{-1.4t}+67872.2e^{-1.3t}-109161e^{-1.2t}\\ +143670e^{-1.1t}-155430e^{-t}+138262e^{-0.9t}-100721e^{-0.8t}+59540.1e^{-0.7t}\\ -28099.4e^{-0.6t}+10297e^{-0.5t}-2786.09e^{-0.4t}+499.369e^{-0.3t}-40.602e^{-0.2t}\\ -3.74085e^{-0.1t}. \end{array}$$

This real Dirichlet series solution *R*_20_(*t*) leads to other solutions *I*_20_, *E*_20_, and *S*_20_ by using (41)-(43). Note that for each *N* the solution *R*_*N*_(*t*) gives the long-run behavior *R*_∞_. In the considered situation, we have *R*_∞_ = 0.997535 which is obtained by solving Eq (34). We also can note here that the constant 0.997535 is the constant term of *R*_*N*_(*t*) for any integer *N*. It can be concluded that under the given initial conditions and parameters in this subsection, the long-run behavior of the SEIR solution is

$$\left(S_{\infty },E_{\infty },I_{\infty },R_{\infty }\right)=\left(0.002465,0,0,0.997535\right).$$

### Error comparison of the SEIR model

**Fig 6:**
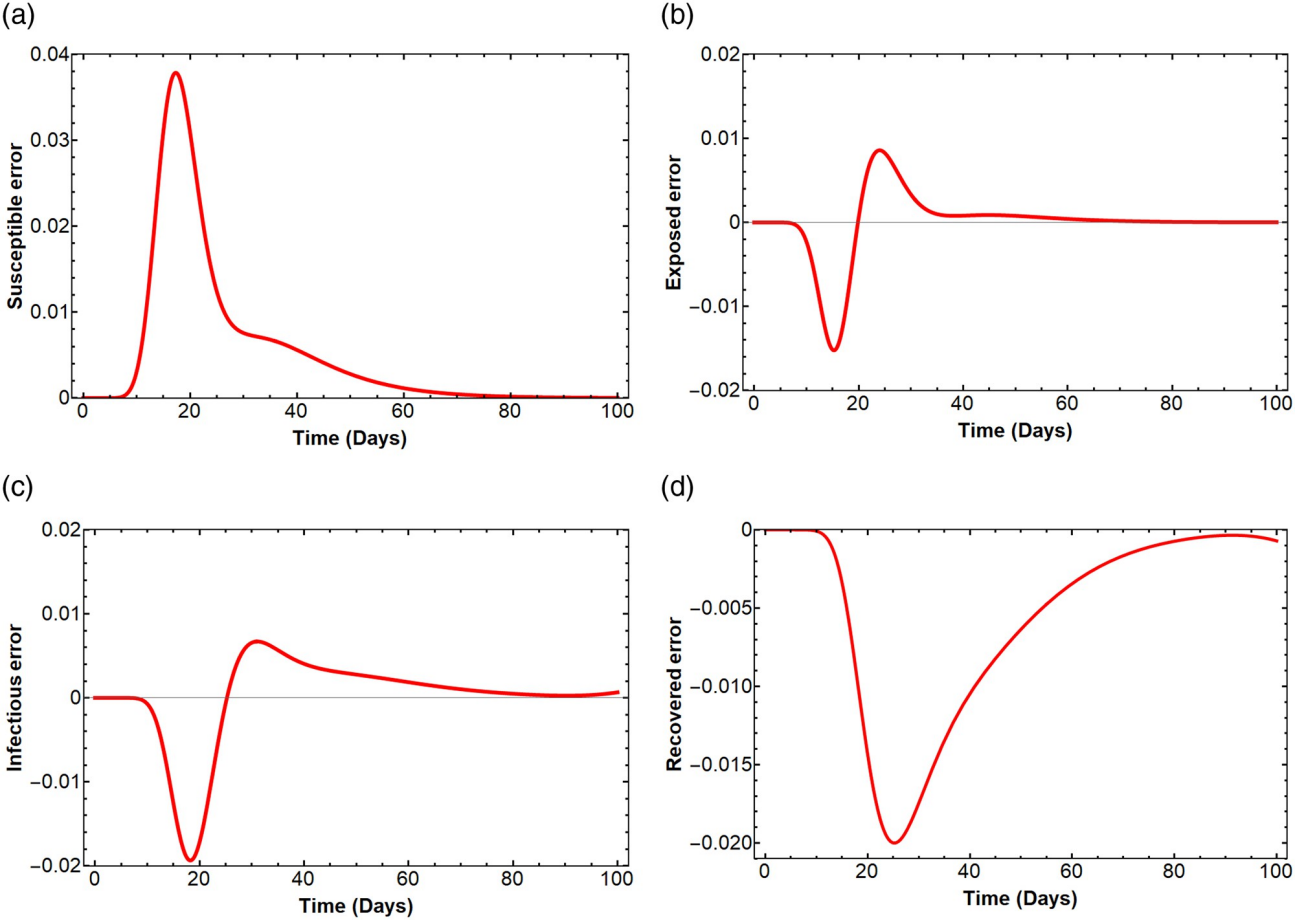
Error of the solutions *S*_20_(*t*), *E*_20_(*t*), *I*_20_(*t*), *R*_20_(*t*) compared with the solutions *S*(*t*), *E*(*t*), *I*(*t*), *R*(*t*) obtained from the RK-4 method.

The accuracy of our method can be observed by investigating the mean square error. Fig 6 shows the errors of *S*_20_(*t*), *E*_20_(*t*), *I*_20_(*t*), *R*_20_(*t*) compared with the numerical solution obtained by the RK-4 method. The figure shows the graphs of the differences *S*_15_(*t*) − *S*(*t*), *E*_15_(*t*) − *E*(*t*), *I*_15_(*t*) − *I*(*t*), and *R*_15_(*t*) − *R*(*t*) at any time *t*. The mean square errors of *S*_20_(*t*), *E*_20_(*t*), *I*_20_(*t*), *R*_20_(*t*) compared with the numerical solutions *S*(*t*), *E*(*t*), *I*(*t*), *R*(*t*) are 0.000111401, 0.0000150904, 0.0000287791 and 0.0000675307, respectively.

## Conclusions

The presented method needs no step-size interpolation and also shows the long-run behavior of the approximate solutions; however, the exact parametric solution (SIR case) and the solution from the RK-4 method (both SIR and SEIR cases) cannot give the long-run behavior of the system since their processes need step-size interpolation. The exact parametric analytical solution (SIR case) presented in [19] does not give the closed-form solution as a function of time and it still needs a numerical method to get the SIR solution at any time *t*. The approximate closed-form Dirichlet series solution can be an alternative direct method to get the approximate solution of the SIR and SEIR models.

The theoretical results in this paper provide a numerical algorithm in order to obtain a numerical solution of the SIR and SEIR models. The derived Dirichlet series approximants obviously preserve the positive property of the SIR and SEIR solutions. The comparisons of the solution graphs shown in Figs 1, 2, 4, and 5 assure that the presented method in this manuscript can be used as an alternative method. The SIR solution in the Dirichlet series form obviously gives the long-run behavior (*S*_∞_, 0, 1 − *S*_∞_) which depends on the given initial conditions and the model’s parameters. As a consequence of the approach to the SIR model, a similar process is used with the SEIR model. The assumed characteristic of the solution *R*(*t*) as a real Dirichlet series leads us to the approximated solutions of the SEIR model. The solutions *S*_*N*_(*t*), *E*_*N*_(*t*), *I*_*N*_(*t*), *R*_*N*_(*t*)) obtained by our method is almost perfectly matched the solution from the RK-4 method. The solutions also obviously preserve the positive property of the SEIR model and carry the long-run behavior (*S*_∞_, *E*_∞_, *I*_∞_, *R*_∞_) which depends on the initial conditions and parameters. The method presented in this manuscript is not only useful for obtaining the SIR and SEIR models but it also can be used in many kinds of nonlinear dynamical systems.

## Supporting information

S1 File Wolfram mathematica codes. There are no raw data used in this paper. All figures in this manuscript are generated by Wolfram Mathematica posted at https://github.com/Kiattisak-Prathom/Mathematica-Codes-for-PLOS-ONE.
(ZIP)

## References

[1] Weiss HH. The SIR model and the foundations of public health. Materials matematics. 2013:0001–17.

[2] Cooper I, Mondal A, Antonopoulos CG. A SIR model assumption for the spread of COVID-19 in different communities. Chaos, Solitons & Fractals. 2020 Oct 1;139:110057. doi: 10.1016/j.chaos.2020.110057 32834610 PMC7321055

[3] Acemoglu D, Chernozhukov V, Werning I, Whinston MD. Optimal targeted lockdowns in a multigroup SIR model. American Economic Review: Insights. 2021 Dec;3(4):487–502.

[4] Wintachai P, Prathom K. Stability analysis of SEIR model related to efficiency of vaccines for COVID-19 situation. Heliyon. 2021 Apr 1;7(4):e06812. doi: 10.1016/j.heliyon.2021.e06812 33880423 PMC8048396

[5] Biswas MH, Paiva LT, de Pinho MD. A SEIR model for control of infectious diseases with constraints. Mathematical Biosciences and Engineering. 2014 Aug 1;11(4):761–84. doi: 10.3934/mbe.2014.11.761

[6] Mwalili S, Kimathi M, Ojiambo V, Gathungu D, Mbogo R. SEIR model for COVID-19 dynamics incorporating the environment and social distancing. BMC Research Notes. 2020 Dec;13(1):1–5. doi: 10.1186/s13104-020-05192-1 32703315 PMC7376536

[7] López L, Rodo X. A modified SEIR model to predict the COVID-19 outbreak in Spain and Italy: simulating control scenarios and multi-scale epidemics. Results in Physics. 2021 Feb 1;21:103746. doi: 10.1016/j.rinp.2020.103746 33391984 PMC7759445

[8] Ragusa MA, Razani A. Weak solutions for a system of quasilinear elliptic equations. arXiv preprint arXiv:2006.05262. 2020 Jun 6.

[9] Goodarzi Z, Mokhtarzadeh MR, Pournaki MR, Razani A. A Note On Periodic Solutions Of Matrix Riccati Differential Equations. Applied Mathematics E-Notes. 2021;21:179–86.

[10] Razani A. An existence theorem for ordinary differential equation in Menger probabilistic metric space. Miskolc Mathematical Notes. 2014;15(2):711–6. doi: 10.18514/MMN.2014.640

[11] Bjørnstad ON, Shea K, Krzywinski M, Altman N. The SEIRS model for infectious disease dynamics. Nature methods. 2020 Jun 1;17(6):557–9. doi: 10.1038/s41592-020-0856-2 32499633

[12] Trejo I, Hengartner NW. A modified Susceptible-Infected-Recovered model for observed under-reported incidence data. PloS one. 2022 Feb 9;17(2):e0263047. doi: 10.1371/journal.pone.0263047 35139110 PMC8827465

[13] Cuspilici A, Monforte P, Ragusa MA. Study of Saharan dust influence on PM10 measures in Sicily from 2013 to 2015. Ecological Indicators. 2017 May 1;76:297–303. doi: 10.1016/j.ecolind.2017.01.016

[14] Duro A, Piccione V, Ragusa MA, Veneziano V. New environmentally sensitive patch index-ESPI-for MEDALUS protocol. InAIP Conference Proceedings 2014 Dec 10 (Vol. 1637, No. 1, pp. 305-312). American Institute of Physics.

[15] Salman SM. On a discretized fractional-order SIR model for Influenza A viruses. Prog. Fract. Differ. Appl. 2017;3(2):163–73. doi: 10.18576/pfda/030207

[16] Das AG, Lahiri BK. Dirichlet series solutions of differential equations. Rendiconti del Circolo Matematico di Palermo. 1984 Sep;33:425–35. doi: 10.1007/BF02844504

[17] Sachdev PL, Bujurke NM, Pai NP. Dirichlet series solution of equations arising in boundary layer theory. Mathematical and computer modelling. 2000 Nov 1;32(9):971–80. doi: 10.1016/S0895-7177(00)00183-7

[18] Laohakosol V, Phuksuwan O, Prathom K. Dirichlet series solutions of generalized Riccati equations. European Journal of Mathematics. 2015 Mar;1:170–85. doi: 10.1007/s40879-014-0021-5

[19] Harko T, Lobo FS, Mak M. Exact analytical solutions of the Susceptible-Infected-Recovered (SIR) epidemic model and of the SIR model with equal death and birth rates. Applied Mathematics and Computation. 2014 Jun 1;236:184–94. doi: 10.1016/j.amc.2014.03.030

[20] Barlow NS, Weinstein SJ. Accurate closed-form solution of the SIR epidemic model. Physica D: Nonlinear Phenomena. 2020 Jul 1;408:132540. doi: 10.1016/j.physd.2020.132540 32362697 PMC7195136

[21] Heng K, Althaus CL. The approximately universal shapes of epidemic curves in the Susceptible-Exposed-Infectious-Recovered (SEIR) model. Scientific Reports. 2020 Nov 9;10(1):19365. doi: 10.1038/s41598-020-76563-8 33168932 PMC7653910

[22] Yıldırım A, Cherruault Y. Analytical approximate solution of a SIR epidemic model with constant vaccination strategy by homotopy perturbation method. Kybernetes. 2009 Oct 16;38(9):1566–75. doi: 10.1108/03684920910991540

[23] Izadi M, Seifaddini M, Afshar M. Approximate solutions of a SIR epidemiological model of computer viruses. Advanced Studies: Euro-Tbilisi Mathematical Journal. 2021 Dec;14(4):203–19.

[24] Srivastava HM, Area Carracedo IC, Nieto JJ. Power-series solution of compartmental epidemiological models. Mathematical Biosciences and Engineering. 2021 Apr 12. doi: 10.3934/mbe.2021163 34198385

[25] Akindeinde SO. A new multistage technique for approximate analytical solution of nonlinear differential equations. Heliyon. 2020 Oct 1;6(10):e05188. doi: 10.1016/j.heliyon.2020.e05188 33088955 PMC7567931

[26] Vazquez-Leal H, Benhammouda B, Filobello-Nino U, Sarmiento-Reyes A, Jimenez-Fernandez VM, Garcia-Gervacio JL, et al. Direct application of Padé approximant for solving nonlinear differential equations. SpringerPlus. 2014 Dec;3(1):1–1. doi: 10.1186/2193-1801-3-563 25332863 PMC4194307

[27] Baker GA, Baker GA Jr, Graves-Morris P, Baker SS. Pade Approximants: Encyclopedia of Mathematics and It’s Applications, Vol. 59 Baker George A. Jr., Peter Graves-Morris. Cambridge University Press; 1996 Jan 26.

[28] Turner LR. Inverse of the Vandermonde matrix with applications. 1966 Aug 1.

[29] Carcione JM, Santos JE, Bagaini C, Ba J. A simulation of a COVID-19 epidemic based on a deterministic SEIR model. Frontiers in public health. 2020 May 28;8:230. doi: 10.3389/fpubh.2020.00230 32574303 PMC7270399

